# “Slowly, Over Time, You Completely Lose Yourself”: Conceptualizing Coercive Control Trauma in Intimate Partner Relationships

**DOI:** 10.1177/08862605251320998

**Published:** 2025-02-23

**Authors:** Kristy Kassing, Anthony Collins

**Affiliations:** 1 La Trobe University, Melbourne, Australia; 2 Rhodes University, Makhanda, South Africa

**Keywords:** coercive control, trauma, intimate partner violence, victim-survivors, violence

## Abstract

Coercive control is a form of violence characterized by patterns of restrictive regulation, including through isolation, threats, psychological manipulation, economic abuse, technology-facilitated control, stalking, and physical and/or sexual violence. While literature has focused on the diverse forms of control imposed by perpetrators of this abuse, few studies have discussed the traumatic impact of coercive control on victim-survivors themselves. Using a theoretical thematic analysis, this study draws upon data from 15 qualitative questionnaires exploring the lived experiences of women as victim-survivors of coercive control in Australia. The first phase of analysis identified the diverse and interconnected methods of coercive control experienced by participants. The second phase found that victim-survivors who have been subjected to these patterns of harm can experience specific emotional trauma, shaped by processes of threat and restraint. This paper, therefore, proposes the concept of “coercive control trauma” to articulate ways in which coercive control in intimate relationships can contribute to the complex and often under-recognized psychological harm experienced by victim-survivors. The concept of coercive control trauma may provide victim-survivors with validation, insight, and an empowering sense of self-understanding during processes of recovery. It may also assist those who are supporting individuals recovering from coercive control by helping to further comprehend the experiences of victim-survivors, and to provide more appropriate forms of support.

## Introduction

It has long been understood that physical and sexual violence characterizes intimate partner violence (IPV) (Kelly & Johnson, 2008). However, many of these relationships are also shaped by patterns of *non-physical* harm—conceptualized as coercive control (Stark, 2007). Coercive control involves repeated incidents that can cause victim-survivors to feel shame, confusion, anxiety, terror, and helplessness (Herman, 2015; Williamson, 2010). For example, such an incident may involve one deliberately taking their partner’s vehicle on a regular basis before work to ensure that they cannot attend, thereby impacting their partner’s employment and collegial relationships. Viewed in isolation, this “unremarkable” event is not likely to be considered abusive. Yet when similar events consistently occur, the cumulative nature of these non-physical harms—working together to gradually create a regime of coercive control—can contribute to a specific experience of psychological trauma.

This paper proposes “coercive control trauma” as a conceptual framework for further understanding the ways in which coercive control can be traumatic for women as victim-survivors. The term is *not* offered as a psycho-diagnostic category, but rather as a lens for clarifying and re-framing the harms caused by coercive and controlling regimes—allowing for further understanding of these incidents and the complex responses of women affected by them. This framework builds upon existing feminist conceptualizations of coercive control, using data from 15 qualitative questionnaires with women who previously experienced coercive control in intimate partner relationships. The notion of coercive control trauma seeks to illustrate and explain how coercive control can be psychologically harmful. Earlier research has emphasized risks flagged by coercive control as a *precursor* for physical and/or sexual harm or intimate partner homicide (see Tyson, 2020), and ways in which it limits the rights of those controlled. The aim of this paper, however, is to extend upon literature by clarifying how coercive control itself is an under-recognized form of violence that can damage one’s sense of self through the ongoing deprivation of liberty and autonomy, and the creation of an environment characterized by pervasive helplessness and fear.

## Background and Context for the Study

### IPV and Coercive Control

IPV can be described as “abusive behavior by a person within an intimate relationship” (Fitz-Gibbon et al., 2018, p. 1). Many IPV victim-survivors identify as women (Australian Institute of Health & Welfare, 2023), yet IPV may be experienced by any individual, including LGBTQIA+ groups and men (Drijber et al., 2013; Walters, 2011). Experiencing IPV has been associated with low self-esteem, symptoms of anxiety and/or depression, economic instability, and housing instability (Candela, 2016; Karakurt et al., 2014; Pitman, 2017; Williamson, 2010). Some victim-survivors of IPV may develop post-traumatic stress disorder (PTSD) or experience symptoms closely associated with PTSD (Levine & Fritz, 2016; Taft et al., 2005). Those who experience prolonged IPV may suffer from complex PTSD (C-PTSD), shaped by more sustained disturbances to one’s well-being and sense of identity (Cloitre et al., 2009; Gallagher et al., 2022; Herman, 2015).

### The Traumatic Nature of Coercive Control as IPV

Coercive control encompasses patterns of predominately *non-physical* harm that can result in “systematic and targeted entrapment” (Crossman et al., 2016, p. 203; Fontes, 2015; Hamberger et al., 2017). Strategies can include psychological abuse, economic manipulation, isolation, stalking, and technology-facilitated control, leading to the development of a regime of domination and monitoring over ordinary daily behaviors (Dragiewicz et al., 2018; Fontes, 2015; Harris & Woodlock, 2018; Kelly & Johnson, 2008; Lammers et al., 2005; Postmus et al., 2020). Threats are often used (Crossman et al., 2016) and may result in sexual violence, physical abuse, or, in some instances, intimate partner homicide (Arnold, 2009; Mitchell & Raghavan, 2021; Pitman, 2017). When such tactics are inflicted repetitively, a “condition of unfreedom” is created, leading to one’s entrapment (Stark, 2007, p. 205). Due to traditional definitions of IPV regarding “crimes to be discrete acts” (Stark, 2007, p. 86), tactics of coercion and control as non-physical harm were not historically understood as IPV, as these acts in isolation were not perceived as rising to the threshold of violence. Coercive control was instead understood as a *context* for physical harm (Crossman et al., 2016) rather than a distinct abusive category itself. Stark’s (2007) theory of coercive control, however, maintains that patterns of non-physical harm are central to most abusive relationships in which men ultimately restrain and deprive women of liberty.

Coercive control can be considered traumatic when conceptualized as a *sustained* regime of harm in an intimate relationship and when its ability to damage the self via continual perpetration of abuse is recognized (Arnold, 2009; Candela, 2016; Stark, 2007). This regime essentially restricts victim-survivors from engagements with external social networks by causing the continuous anticipation of potential negative and/or unpredictable consequences from the aggressor, creating a system of risk and isolation alongside affection and security (Herman, 2015). Diverse interpersonal, social, and cultural factors that contribute to the development of attachment styles have also been found to affect the dynamics of the traumatic bond that develops between adult perpetrators and victim-survivors (Bond & Bond, 2004; Buchanan, 2013; Dutton & Painter, 1993; Dutton & White, 2012; Park, 2016). Here, the way in which the relationship is the source of both threat/danger *and* reward/security, paired with the turbulence of alternating idealization and denigration common in abusive relationships, can intensify enmeshment and dependency (Herman, 2015; Logan, 2018; Park, 2016). This occurs in such relationships as “domestic captivity” (Herman, 2015, p. 74), via one’s actions being continuously monitored in an environment of uncertainty and fear (Williamson, 2010).

### Psychological Trauma and Coercive Control

A large body of literature has discussed psychological trauma as relating to the clinical diagnosis of PTSD (Davidson, et al., 1991; Regel, 2010; van der Kolk, et al., 1999). PTSD is today described as the experience of “one or more intrusion symptoms associated with the traumatic event(s), beginning after the traumatic event(s) occurred”—event(s) may have been experienced, witnessed, or learned of (American Psychiatric Publishing, 2013, pp. 271–272). Herman (2015) also emphasizes the ways in which hyperarousal, intrusion, and constriction characterize PTSD. Understandings of PTSD as caused by exposure to a *singular* distressing event were later challenged by theories of C-PTSD, recognizing that trauma can also be experienced following exposure to multiple or ongoing distressing events for extensive time periods (Root, 1989).

Root’s (1989) conceptualization of “insidious trauma” extends upon theories of PTSD and C-PTSD by demonstrating, through a critical feminist framework, that trauma is not only the result of singular or even multiple harmful incidents, but may also be a consequence of ongoing *covert* harms. For example, these may involve forms of social and cultural oppression and/or the continuous *threat* of violence experienced by societies, communities, and individuals—particularly girls and women—in which conditions of terror and risk characterize “ordinary” life in almost unnoticeable ways. This concept also recognizes the impact of private violations commonly suffered by women on an interpersonal and socio-cultural level that are “not outside the range of human experience” (Brown, 1991, p. 120). These may include, for instance, incest, rape, harassment, and/or merely the pervasive *fear* of such events being experienced by women in high-risk situations (Brown, 1991).

Brown’s (1991) and Root’s (1989) contributions are significant to feminist understandings coercive control because they emphasize that trauma is not only caused by exposure to one or more distressing events leading to intrusive symptoms. Rather, seemingly “ordinary” harms repeated in a social context of normalized gender-based violence, as well as the ongoing *anticipation* of such events in environments of high-risk, can also contribute to trauma experienced by women (Brown, 1991; Herman, 2015; Root, 1989). Coercive control, involving patterns of predominantly non-physical harm, may therefore cause significant distress due to the cumulative impact of strategies enacted in intimate relationships that are not explicitly harmful, but which have the ability to become women’s everyday reality of constraint and threat (Herman, 2015; Stark, 2007; Williamson, 2010). Brown’s (1991) and Root (1989) arguments have since been expanded upon by critical feminist and trauma scholars, particularly in regard to the impact of coercive control (Anderson, 2009; Crossman & Hardesty, 2018; Herman, 2015; Stark, 2007). This paper, therefore, proposes “coercive control trauma” as a conceptual tool to describe and further demonstrate *how* this process may be traumatic—not only as a precursor to potential physical abuse and/or intimate partner homicide, but as a standalone form of insidious psychological violence itself.

## Study Description

### Theoretical Frameworks

A feminist, trauma-informed, and victim-centered theoretical perspective was adopted and sought to center participants’ structural positioning in relation to gender identity, highlighting the “validity of women’s experiences” in an IPV context (Driscoll & McFarland, 1989, p. 187). The issue of note is that structural relations of inequality (patriarchal or other) may place one at risk of psychological, economic, technology-based, and/or physical exploitation, though this is not necessarily reducible to social group membership. The trauma-informed and victim-centered lens aimed to acknowledge the ways in which victim-survivors may experience distress following coercive control (Herman, 2015). This also assisted in preventing re-traumatization by placing participants’ well-being at the center of the research (Bowen & Murshid, 2016; Substance Abuse and Mental Health Services Trauma and Justice Strategic Initiative, 2014). While the trauma-informed lens is generally ethically suitable for studies involving victim-survivors, its tendency to “reproduce problematic assumptions about how victim-survivors ‘should’ experience the impacts of violence” (Mortimer et al., 2021, p. 146)—often grounded in Euro-centric perspectives of trauma and carceral responses to IPV—must be highlighted. To avoid this, the researchers validated the diversity and complexity of participants’ responses and reflections at all stages of the research. This allowed for differing interpersonal, social, and cultural circumstances to be considered—all of which can contribute to post-traumatic reactions that may fall outside of Western diagnostic criterion and/or may challenge responses that criminal legal systems predict victims will have (Atkinson, 2020; Brown, 1991; Herman, 2015).

### Data Collection and Analysis

Ethics approval was granted by the La Trobe University Human Ethics Committee [HEC20330]. Fifteen women with previous lived experience of coercive control were recruited from a large online survivor and advocacy support group, and qualitative semi-structured questionnaires were distributed (see Appendix). Self-selective sampling techniques were used due to project time constraints and to ensure that data gathered from online community members were as comprehensive as possible, despite the small final sample size. Self-selective sampling techniques helped ensure that participants were voluntarily willing to provide responses to questions. Trauma-informed research practices were adopted at all stages of the research and a risk assessment plan was adhered to. Participants’ names were replaced with pseudonyms, and all identifying information was removed or anonymized.

Theoretical thematic analysis was then used to interpret and analyze data (Boyatzis, 1998; Braun & Clarke, 2006; Pitman, 2017). This was due to the sensitive nature of the study, the small non-representative sample, and selected theoretical frameworks. Theoretical thematic analysis is a widely used method involving “analyzing and reporting patterns” (Braun & Clarke, 2006, p. 79), allowing for analysis based upon pre-existing conceptual frameworks. This can also assist in the interpretation of personal or sensitive testimonies, such as those shared by participants with lived experience of IPV (Ho et al., 2017; Pitman, 2017). Following this method, the first author commenced in-depth readings of data and assigned codes to capture all elements of reported experiences. Codes were then grouped into various themes through an iterative process of (re)combining, (re)organizing, and (re)refining until coherence and consistency existed within and across themes (Attride-Stirling, 2001; Braun & Clarke, 2006; Miles & Huberman, 1994). The final stage moved beyond presentation of themes to the development of a framework for understanding the traumatic impact of coercive control. Here, themes were further interrogated through critical trauma and feminist theory (see, e.g., Brown, 1991; Herman, 2015; Root, 1989; Williamson, 2010). Deductive readings through theoretical frameworks were conducted by both authors to assess whether themes supported the conceptual elements of theories (Attride-Stirling, 2001; Roberts et al., 2019). As common in much interpretivist research (Ho et al., 2017), not all themes aligned with theories in a mutually exclusive manner, leading both authors to further (re)construct, review, and refine iterations of the framework until consensus was established that most appropriately supported the data and analysis. Digital coding programs were not used.

## Research Outcomes and Discussion

**Figure 1.**
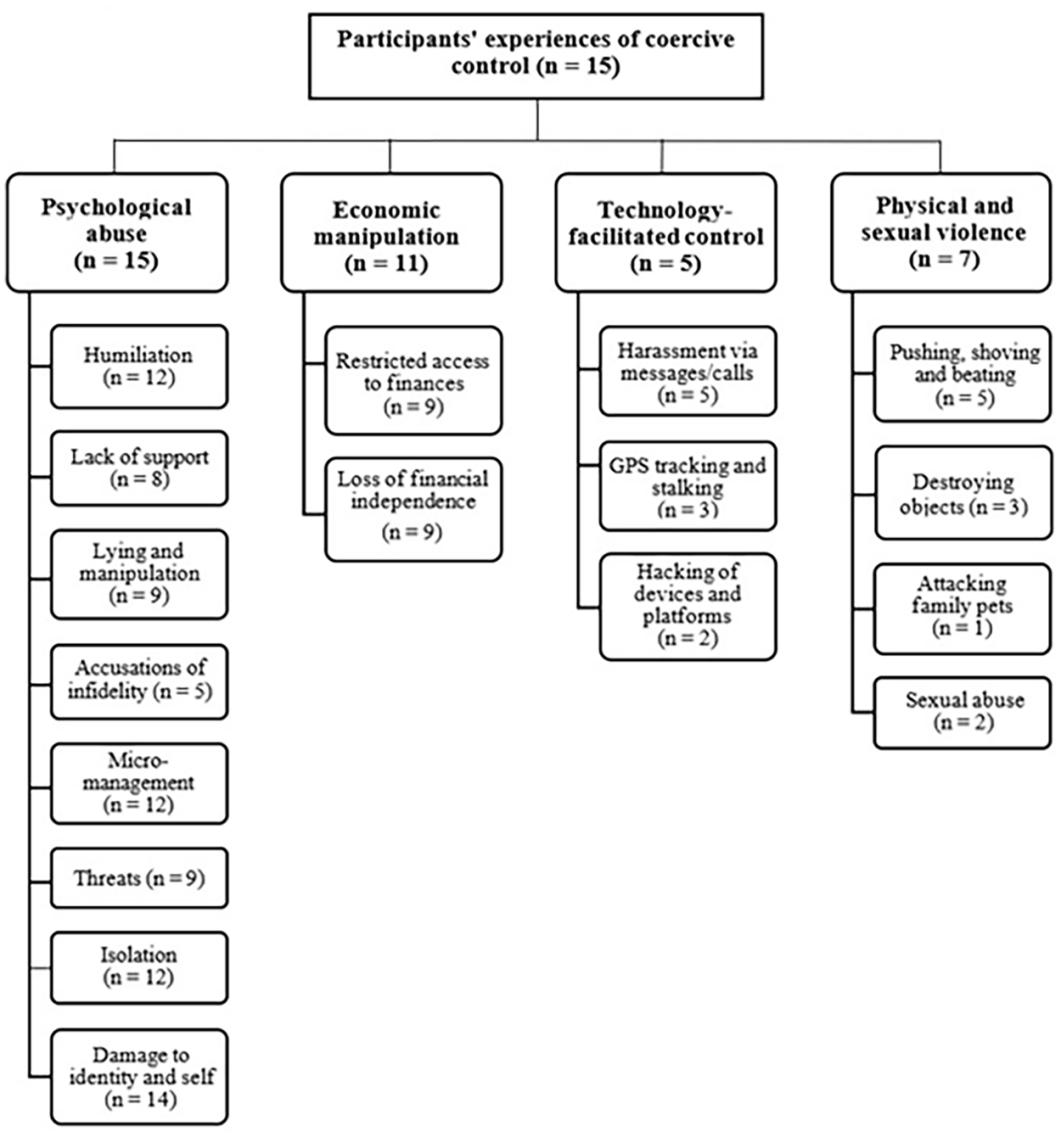
Developed thematic map, showing main themes and sub-themes.

**Figure 2.**
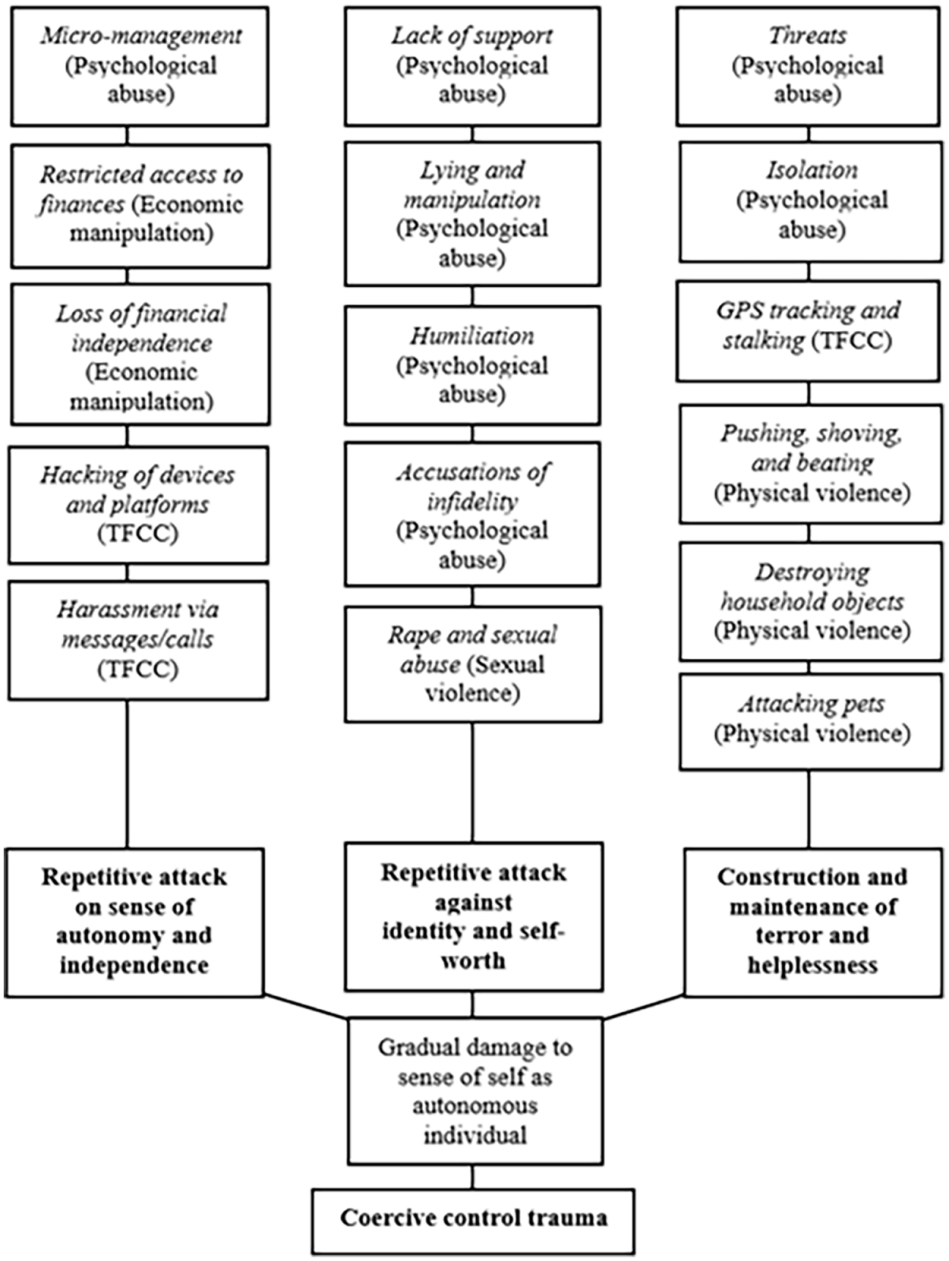
Developed thematic map, demonstrating the coercive control trauma framework.

Four main themes and various sub-themes were identified during the first analysis phase. This focused on the forms of abuse that participants (*n* = 15) reported having experienced as coercive control, including: psychological abuse (*n* = 15), economic manipulation (*n* = 11), technology-facilitated control (*n* = 5), and physical and/or sexual violence (*n* = 7). These themes are presented below in a developed thematic map (Figure 1). The following sections now provide a descriptive overview of each theme, characterizing participants’ experiences of coercive control. These also represent the strategies of abuse that contributed to participants’ experiences of *coercive control trauma* (Figure 2). Both thematic maps arose from the discourse of women in the sample who shared a common structure of lived experience, with each map visually representing the data coding and analysis through which the coercive control trauma framework developed. Themes presented are neither exclusive nor comprehensive categories. Due to the unpredictable and insidious nature of coercive control, each theme that represents a component of abuse that may co-exist and closely interconnect/overlap without prescribed order or predictability.

### Psychological Abuse

#### Humiliation, Manipulation, and Lack of Support

All participants (*n* = 15) experienced psychological abuse. This highlighted perpetrators’ tendency to degrade participants as women via humiliation reflecting rigid gendered ideals, such as “not meeting his needs (sexually)” and “not cleaning well enough.” Participants were humiliated and manipulated, and told they were “sexually unattractive,” “ugly,” or that they failed to fulfill domestic duties. For example, Kiera recalled:

(. . .) If I needed to go from work to family functions, he always took my car just before I would need to leave. He would say he’d be five minutes, but then be gone for hours. I lost more than one job for being late to work on many occasions because of this. (Kiera)

Lack of support was described as “a form of coercive control.” As Jeanette stated: “there was a total lack of support on any level, and the result almost broke my spirit.” Dianne explained:

They would put me down, sneer at me, and blame me. I was told I was ‘materialistic’ when I was standing there in rags, no makeup, no hairdo, no proper shoes, and scraping every cent I could just to put food on the table. This made me not want to go out due to the shame I felt about my appearance. (Dianne)

#### Accusations, Lying, and Micro-Management

Psychological abuse often involved “hurtful” accusations of infidelity, lying, and micro-management. Demi stated: “if I spoke too long to one of his male friends, or if I smiled too much or laughed, I was the ‘slut’ flirting with men just to annoy him.” Dianne discussed: “. . . the lies escalated through the roof. He would ring the police to tell them I was bashing him—I didn’t lay a finger on him. The lies were very alienating. . ..” Further, micro-management was used to undermine and restrain. As noted by Sophia: “he constantly tried to micro-manage my career (. . .) At one point, he refused to let me work more than three hours per day.”

#### Threats

Threats of abandonment, violence, self-harm, and/or murder were tactics used to maintain control while contributing to a sense of fear and helplessness. Charli, for instance, explained that her partner would threaten to self-harm if she “did not pander to his way of thinking or demands.” As Alison recalled:

He said if I left, he would chop me up and put me in a cray pot. After separation, he breached his intervention order and attempted to come and kill me (. . .). (Alison)

#### Isolation

Isolation contributed to participants feeling they had “no rights, independence, or freedom.” They were made to feel “guilty” for interacting with social networks, or were isolated by being forced to physically relocate. Sophia, for example, “gradually stopped making arrangements” with friends because “it was too cumbersome always justifying why I liked them, despite his disapproval.” Ashley recalled her partner would demand to move locations unexpectedly, and “he would always try to alienate me from my friends and family.”

#### Damage to Self, Identity, and Sense of Agency

Participants’ understandings of their identities were progressively disrupted due to continuous psychological abuse, yet remaining in the relationship also became necessary for survival. Ashley stated: “these little things all add up, and slowly, over time, you completely lose yourself, your independence, and your desire to make choices.” Further, Alison noted:

I began to lose all my self-worth. I still really struggle with putting myself first, and with speaking my mind (. . .) I have trouble making decisions for myself in fear it will be the wrong choice. I was too scared and embarrassed to tell anyone what was going on, so I just painted on my happy face and continued to try to keep surviving. (Alison)

### Psychological Abuse as Central to Coercive Control

The findings reaffirm psychological abuse as a component of coercive control in the sense that subtle patterns of psychological harm entrapped participants in an environment of “domestic captivity” (Devries, et al., 2013; Herman, 2015, p. 74; Stark, 2007). This strengthened participants’ dependency on their abusers (Park, 2016), contributing to feelings of being unable to trust or act upon their judgment due to the sustained manipulative environment (and risk of further abuse) (Fontes, 2015; Herman, 2015; Williamson, 2010). Participants also experienced reactions associated with post-traumatic hyperarousal, intrusion, and constriction (Herman, 2015; Taft et al., 2005). Hyperarousal was reflected in statements such as: feeling “afraid,” “sick with dread,” and “I was walking on eggshells.” Intrusion and construction was reflected in feeling “trapped,” “scared,” “zombie-like,” “dead,” and “you want to kill yourself to avoid seeing the abuser.” Psychological abuse caused damage to participants’ sense of self via the deprivation of autonomy (Candela, 2016; Crossman et al., 2016; Herman, 2015; Kelly, 2010), while being directed toward participants social positioning as women (Arnold, 2009; Lawson, 2012). Adopting such tactics to control and reinstate gendered norms indicated that harmful heteropatriarchal attitudes were reproduced (Anderson, 2009; Fontes, 2015; Williamson, 2010). As Dianne recalled: “I was told I was ugly and sexually unattractive, and that they didn’t know why they married me.” This increased feelings of entrapment and reflected ways in which participants were vulnerable as women due to structural gender inequality (Arnold, 2009; Stark, 2007).

### Economic Manipulation

#### Restricted Access to Finances

Economic manipulation was a form of coercive control experienced by most participants (*n* = 11). Participants were deprived of access to their salaries, causing them to feel “boxed in and pushed into a corner.” Economic manipulation ensured that “it was very hard to release” from perpetrators and contributed to psychological abuse, further illustrating the presence of rigid heteropatriarchal beliefs: “when I stood up against him and opened a private account, he really freaked out and started psychologically abusing me (. . .) he said I belonged to him, and that I was selfish and wasteful” (Whitney).

I couldn’t afford to buy food and was scared of being ripped down about not having money to afford his lifestyle, so I stopped eating (. . .) I became sick and thin, and then he’d compliment me for looking like a model. He’d say it was the only reason he wasted his time with me and would then joke about me being cheap to feed. (Louise)

Perpetrators’ financial control also resulted in participants being prevented from social activities or travel. This heightened feelings of isolation and powerlessness by ensuring that they were unable to receive support external to their relationship. Jeanette explained:

We had no freedom. I had no financial independence and thus no way to formulate an escape plan. I had no money and nowhere to go. I did not believe there was any way I could leave and provide a safe home to support my child. (Jeanette)

#### Loss of Financial Independence

Economic manipulation left participants with little choice but to remain financially dependent on their partners. Participants were entirely prevented from accessing finances or were forced to “beg” for support, exacerbating symptoms of trauma (Herman, 2015; Stark, 2007). This reflected the ways in which perpetrators were inclined to abuse areas of participants’ lives (in this instance, control of finances) representing women’s historically “inferior” social status (Adams et al., 2008; Arnold, 2009), and demonstrating how socio-cultural beliefs relating to men’s entitlement to women can facilitate such abuse (Hunnicutt, 2009; Postmus et al., 2020; Yodanis, 2004).

### Technology Facilitated Control

#### Harassment, GPS Tracking/Stalking, and Hacking

Participants (*n* = 5) were also subjected to technology-facilitated control (Dragiewicz et al., 2018; Rogers et al., 2022) involving micro-management, harassment, and technological stalking. These harms caused participants to feel “constantly watched, monitored, judged, and ridiculed.” As Jennifer stated: “he adopted technological surveillance through apps and demanded to know my passwords.” Participants’ devices and social media platforms were also regularly breached. Further, Alison explained that her partner would track her phone and continually question her location: “(. . .) I was constantly made to feel like I was lying. He logged onto my accounts to keep tabs on me and to see who I was talking to, but he would also chat to people online and pretend to be me.” Technology-facilitated control contributed to participants’ deprivation of autonomy by increasing their sense of powerlessness. Devices and social media platforms provided tools through which coercive control could be inflicted at any time and in any space, demonstrating perpetrators’ omnipresence and participants’ entrapment (Arnold, 2009; Dragiewicz et al., 2018; Harris & Woodlock, 2018; Rogers et al., 2022).

### Physical and/or Sexual Violence

#### Pushing/Shoving/Beating, Destroying Objects, Attacking Pets, and Sexual Abuse

Certain participants (*n* = 7) experienced physical and/or sexual violence. Perpetrators often used physical violence in ways that targeted participants’ property, vulnerabilities, or insecurities. Dianne noted: “He would smash things that were precious to me (. . .) because he *knew* they were precious to me. That partner would push me over and say it was ‘not domestic violence’ to push people over.” Jeanette recalled that her partner became enraged and would “slam draws and doors, knowing I was very jumpy,” and would “shove past me in dooways (. . .) he slammed cutlery into draws—something he knew I found disturbing.” Two participants (*n* = 2) were subjected to rape and sexual abuse. Alison disclosed she “felt like a used sex slave,” while Lucinda explained: “the rapes I endured previously had become more frequent and violent.” Due to the sporadic yet repetitive nature of sexual and/or physical violence, and its perpetration by a partner with whom positive experiences and resources were shared, this violence was particularly distressing (Arnold, 2009; Herman, 2015; Pitman, 2017). Perpetrators’ inclination to inflict harm through use of control in traditionally gendered ways was evident via sexual abuse (Williamson, 2010; Yodanis, 2004), reaffirming that women may experience such abuse as gender-based IPV embedded in patterns of non-physical violence (Hamberger et al., 2017, p. 2; Mitchell & Raghavan, 2021).

#### “Coercive Control Trauma”: Impact of Coercive Control as Psychological Violence

The infliction of non-physical and physical harm enabled perpetrators to maintain control while reducing participants’ ability to function autonomously, causing trauma characterized by terror, helplessness, and entrapment. The findings also indicated that harmful gendered ideals were reinforced in participants’ relationships, reaffirming that coercive control is facilitated by structural gender inequality. The following sections now discuss the framework of “coercive control trauma” to further understand the psychological impact of coercive control. Here, the second analysis phase involved the organization of themes guided by models of PTSD and C-PTSD framed by critical feminist and trauma theory (see, e.g., Brown, 1991; Herman, 2015). This revealed that coercive control trauma can be characterized by three processes or covert “attacks” against: (a) the victim-survivor’s autonomy and independence; (b) identity and self-worth; and (c) are facilitated via an environment shaped by terror and helplessness. These attacks can encompass psychological abuse, economic abuse, technology-facilitated coercive control, and physical or sexual violence—the persistent monitoring of movements, decisions, and even thoughts is exercised via these patterns. For instance, engagement in seemingly ordinary acts of autonomy became psychologically dangerous, and increasingly needed to be abandoned for participants to achieve a minimal baseline of safety. The cumulative nature of these processes characterizes coercive control trauma, which and has the potential to damage to the victim-survivor’s identity by reducing their ability to trust and act upon their perception of reality in the abusive environment.

### Attack Against Autonomy and Independence

The first element of coercive control trauma was related to harms that sought to attack participants’ autonomy and independence, shaped by psychological abuse, economic manipulation, and technology-facilitated control. Infliction of these non-physical abusive tactics gradually diminished participants’ ability to act and live autonomously with integrity and control over their lives. For instance, micro-management ensured that participants’ decisions and actions, regardless of how insignificant, were controlled and/or at least influenced by perpetrators. This was compounded by loss of financial independence, hindering participants’ ability to make ordinary decisions and affecting their well-being. Jeanette, for example, explained that when she needed to purchase personal items, she was prohibited from doing so because her abuser maintained control of finances and would humiliate Jeanette if she asked for money. The ongoing *fear* of experiencing further shame or potentially physical violence as “punishment” for requesting money contributed to Jeanette being deprived of her autonomy while experiencing increased helplessness. Technology-facilitated control was also demonstrative of perpetrators’ need to monitor and micro-manage even when physically absent. This caused further damage to participants’ sense of independence by ensuring that they were prevented from choosing when or how to contact their social circles via technology, increasing isolation and affecting participants’ belief in their right to maintain a healthy level of independence.

### Attack Against Identity and Self-Worth

Forms of abuse that enabled perpetrators to attack victim-survivors’ identities and self-worth characterized the second element of coercive control trauma. Feeling inadequately supported, alongside being regularly manipulated, lied to, humiliated, and facing accusations of infidelity, contributed to the gradual altering of participants’ perception of their identities as individuals with value external to their relationships, as Ashley described: “. . .whenever we would walk somewhere, I had to be 45 degrees behind him and to the side. He would occasionally ‘test’ this by swinging his arm back to ensure I was walking in this ‘right spot,’ and he would click his fingers at me if I diverted from this space.” This incident is merely one example of the ways in which repetitive humiliation and micro-management led Ashley to feel that her worth as an autonomous adult, with the ability to demonstrate independence, had been stripped from her—her autonomy was so significantly undermined by seemingly “harmless” acts on a regular basis that it simply became too dangerous to express resistance. Additionally, sexual violence experienced by certain participants contributed to the deterioration of worth by exacerbating entrapment and a sense of helplessness. This occurred alongside psychological control and other manipulative tactics, inflicted unpredictably, and further degrading participants as women to deny them of dignity.

### Construction and Maintenance of Terror and Helplessness

The third element was related to perpetrators constructing and maintaining an environment of terror and helplessness in which victim-survivors were forced to survive. Perpetrators enforced regimes of terror and helplessness that required victim-survivors to endure continual fear. This predominantly involved tactics of psychological abuse, technology-facilitated control, and physical violence. Threats, often rationalized as “responses” to provocations, caused participants to feel at fault and undermined their sense of trust in perceptions of reality while fostering self-blame. Isolation further contributed to terror by removing or restricting social supports, making perpetrators the sole source of validation and security. Tracking and stalking through technology further compounded control, while restricting external support access even when perpetrators were physically absent. For some participants (*n* = 7), physical violence played a significant role in shaping an environment of helplessness. Physical aggression affirmed perpetrators’ domination, reinforcing a sense of captivity (Herman, 2015, p. 74). Some participants felt “deserving” of violence due to perpetrators framing any minor disobedience as an apparent justification for harm. This resulted in heightened feelings of helplessness, undermining participants’ ability to act upon perceived danger due to ongoing threats of harm. These combined strategies entrenched participants in a state of captivity created by the perpetrator, whereby alternating and unpredictable acts of love/terror severely restricted their autonomy and caused feelings of perpetual fear.

## Accumulation of Damage to the Self: Coercive Control Trauma

The coercive control trauma framework illustrates the psychologically harmful nature of coercive control in the context of IPV. This demonstrates the significance of coercive control not only as a risk factor for physical or sexual violence and/or intimate partner homicide, but also because of its ability to insidiously damage one’s sense of identity and well-being. Victim-survivors may experience coercive control trauma due to such abuse encompassing interlinked strategies that work to inflict three covert psychological attacks. These can threaten the victim-survivor’s autonomy and independence, identity, and self-worth and are facilitated by the perpetrator’s ability to foster an environment of terror and helplessness. Experiencing these processes may result in victim-survivors suffering coercive control trauma, which is not the result of a single distressing event, but instead a consequence of subtle and *sustained* harms in a relationship that also provides security and support. The persistent undermining and invalidation of the victim-survivor’s reality, and erosion of one’s ability to rely on perceptions of danger, alongside a loss of trust in decision-making capacity due to the persistent *threat* of harm, are indicative of damage to the once autonomous self. It is the impact of these patterns that may each individually appear “unimportant,” yet work together to facilitate coercive control trauma. This is damaging in itself and heightens the risk of physical and/or sexual harm, as the victim-survivor is isolated from broader social networks and their ability to exercise autonomy is psychologically obstructed.

## Diversity Limitations and Pathways for Future Research

The study presented several limitations while highlighting opportunities for additional research. One major limitation was the small sample size due to the qualitative nature of the study and use of purposive and self-selected techniques. While these allowed for the recruitment of 15 women who were enthusiastic to provide substantial testimony on shared experiences of coercive control, future studies could seek to explore the perspectives and experiences of a broader demographic, such as LGBTQIA+ victim-survivors, refugees, and/or elderly individuals. In Australia, specifically, the views of Aboriginal and Torres Strait Islander peoples who have experienced coercive control interpersonally *and* structurally at the hands of the carceral state require increased attention (Wangmann, 2020). Further, it was beyond the scope of this study to examine how characteristics such as race, class, religion, and migrant/refugee status may have contributed to victim-survivors’ perspectives and the importance of such factors in shaping support interventions for those belonging to minority groups (see Jones & Anyieth, 2023).

## Conclusion

Characterized predominantly by patterns of non-physical harm, coercive control is a form of IPV that has the potential to damage one’s sense of self due to encompassing psychological abuse, economic manipulation, technology-facilitated control, and physical and/or sexual abuse. Following the analysis of 15 victim-survivor testimonies, this study has proposed the notion of “coercive control trauma” as a conceptual tool for understanding the traumatic impact of coercive control in abusive relationships. This term is not suggested as a psycho-diagnostic category, but as an interpretative framework for clarifying and re-framing why those who experience coercive control in relationships of structural inequality may experience significant emotional trauma. The concept may affirm victim-survivors and invoke a sense of empowerment by providing a lens through which the impact of diverse harms suffered can be acknowledged and described. It may also assist victim-survivors during processes of recovery that involve reclaiming agency and autonomy. Further, the concept of coercive control trauma may be of importance to clinicians, domestic and family violence service providers, or legal practitioners assisting individuals who have experienced such abuse.

## Appendix: Semi-Structured Participant Questionnaire

NOTE: As requested by the La Trobe University Human Research Ethics Committee, a definition of coercive control (Q3) was included in the participant questionnaire.

1. Are you finding it helpful being a member of an Australian domestic violence victim-survivor support group on Facebook? In what ways are you finding this beneficial?
2. Besides being a member of an online Facebook support group for victim-survivors, what other things are you finding helpful at the moment for your personal healing or recovery?
3. Evan Stark (2007, p. 198) describes coercive control as repeated behaviors and actions in an intimate relationship, forming “patterns of dominance that entrap partners and make them subordinate.” Common tactics used by perpetrators may include: humiliation, shaming, intimidation, emotional blackmail, isolation, restricted access to financial resources, technological surveillance, and micro-management. What were some of the biggest challenges or difficulties you experienced during your own relationship? When reflecting on these experiences, in what ways did you feel you lacked independence or freedom?
4. Coercive control, specifically, is not currently considered to be a crime in Australia. How do you think we can better assist other women who are experiencing coercive control? What do you think should be done to address the issue of coercive control?
5. If you have previously used a domestic violence support service in Australia, what was your experience like using this service? How do you think this service could be improved to better support other victim-survivors of coercive control?
6. Do you have any other thoughts about the issue of coercive control that you would like to share?
